# Reparative Dentistry: Possibilities and Limitations

**DOI:** 10.1007/s40496-018-0191-1

**Published:** 2018-09-15

**Authors:** Igor Robert Blum, Mutlu Özcan

**Affiliations:** 1 King’s College Hospital & King’s College London Dental Institute, Division of Primary Dental Care and Maurice Wohl Dental Centre, Department of Restorative Dentistry, University of London, Bessemer Road, Denmark Hill, London, SE5 9RS UK; 2 Division of Dental Materials, Center for Dental and Oral Medicine, Clinic for Fixed and Removable Prosthodontics and Dental Materials Science, University of Zurich, Zurich, Switzerland

**Keywords:** Adhesion, Minimally invasive dentistry, Repair, Restorative dentistry

## Abstract

**Purpose of Review:**

Defective dental restorations are amongst the most common encounters in general dental practice. Replacement of defective restorations is often costly and commonly results in the sacrifice of sound tooth structure, thereby compromising the vitality of the dental pulp, potentially resulting in the acceleration of the restoration cycle and premature loss of the restored tooth. With advances in adhesive dentistry, ‘reparative dentistry’ is becoming an important area of minimally invasive dentistry. This article highlights the detrimental biological effects of restoration replacement and provides an overview of current knowledge and understanding of restoration repair as a safe and effective alternative approach to replacement.

**Recent Findings:**

The literature reviewed showed that a growing body of evidence from clinical studies indicates that repaired restorations have similar survival outcomes in patients with low and medium caries risk compared to replaced restorations and are clinically acceptable over a 12-year follow-up of clinical service. Teeth with repaired restorations are less likely to require aggressive interventions such as endodontic treatment or extraction compared to those with replaced restorations.

**Summary:**

Repair options should be carried out wherever possible as minimally interventional procedures in order to increase the longevity of the remaining part of the restoration and the restored tooth unit. Restoration replacement should be considered as the last resort when there are no other viable alternatives.

## Introduction

Owing to hostile conditions in the oral environment, dental restorations commonly suffer deterioration and degradation in clinical service over time. The presence of localised defects in direct restorations and those with the clinical diagnosis of localised secondary caries are amongst the most frequent clinical observations encountered by general dental practitioners [1, 2]. The replacement of restorations constitutes about half of the work performed by general dental practitioners in their practices [3]. In fact, this ‘drill-and-fill’ approach to the management of defective restorations, especially those that exhibit minor imperfections, may be regarded as over-treatment where large portions of the restorations are clinically and radiographically free of failures [4]. The complete removal of defective yet serviceable restorations is based on a flawed mechanistic rather than scientific style of operative dentistry [5]. The result is the removal of the entire restoration and progressive cavity enlargement that often leads to acceleration of the restorative cycle, through the unnecessary removal of intact tooth structure in locations often distant from the site of restoration deterioration, accompanied with the potential for repeated insults to the pulp with increased risk of pulp fatality [5–9]. A further detrimental consequence of the restoration replacement approach includes misuse of patients’ time, resources and tolerance to accept interventive dental care [5, 8, 9].

Whilst some restorations will inevitably require replacement, employing adhesive procedures, many deteriorating yet serviceable restorations may be given extended longevity through repair procedures, especially if the defect is localised and accessible. Reparative dentistry through the application of repair options using surface conditioning methods and resin-based composite materials extends the longevity of the remaining part of the restoration and of the restored tooth by avoiding unnecessary sacrifice of the healthy tooth structure. Thus, today, due to the advances in adhesive technologies performing repair procedures (i.e., partial replacement of a restoration that presents no clinical or radiographic evidence of failure) chairside, using resin composite materials, is considered as an integral part of minimally invasive dentistry.

The repair of a defective restoration could already start with refurbishment—a procedure that should normally pre-empt and delay repair, let alone replacement. Refurbishment procedures typically involve removal of overhangs, surface recontouring, removal of discolouration and smoothening or glazing of surface, including sealing of pores and small gaps, without adding new restorative material except glaze or bonding. Yet, decision making remains one of the key factors whether or not to contemplate any repair action.

## Decision Making and Rationale for Repair

The diagnosis of a defect in a restoration, and the subsequent clinical decision making whether or not to repair, is often based on visual, tactile and radiographic examinations. The most commonly cited defects encountered in direct dental restorations include (1) secondary caries as diagnosed clinically, (2) marginal defects, (3) marginal discoloration and staining, (4) incorrect shade of the restoration, (5) bulk discoloration, (6) bulk fracture of the restoration, (7) fracture of adjacent tooth issue and (8) wear of the restoration [5, 8, 9].

The decision making in respect of ‘defective’ restorations is a critical step in treatment planning, especially given the emerging body of evidence demonstrating the value and effectiveness of procedures to repair restorations that have suffered some form of typically limited deterioration in clinical service [10]. The recognition of one or more limited defects in a restoration does not necessarily mean that the restoration has suffered irreversible damage to the extent that it requires immediate replacement. Most defects in restorations, other than those caused by fracture, develop gradually over extended periods of time, providing the clinician with an opportunity to address the causation of the problem and undertake some form of minimal intervention treatment to correct the defect and thereby extend the longevity of the restoration [11]. Minimal intervention treatment may include repair of the defects, especially if the defects are localised and accessible, or simply refurbishment of the restoration, if the defects are superficial [7–9].

## Teaching

Between 2002 and 2017, 12 surveys were published on the teaching of the repair of resin composite restorations in various countries [4, 12••, 13–22], and one paper reported on the opinion of manufacturers producing dental restorative materials [23]. Owing to the increasing trend at dental schools in numerous countries in favour of the placement of resin composite over amalgam restorations [24–27], all surveys focused on direct composite restorations. Whilst two decades ago, replacement was more common, a recent systematic review and meta-analysis on teaching restoration repair has concluded that the vast majority of dental schools teach restoration repairs [28•]. Surveys investigating the attitudes and experiences of dentists regarding the repair of defective direct restorations in the UK, Germany, Greece and in the Canton of Zürich, Switzerland, reported wide acceptance of the concept of restoration repair amongst dental practitioners and patients [29–32].

## Advantages of Repair

The advantages of repairing restorations with localised defects include:

- Preservation of existing tooth structure and tooth vitality
- Increased longevity of the remaining restoration
- Increased longevity of the restored tooth unit
- Reduction of potentially harmful effects on the dental pulp
- Reduction in treatment time
- Reduced costs to the patient
- Good patient acceptance
- Avoidance of the need for local anaesthesia when repair is limited to a small area.

## Criteria for Repair

A number of factors must be considered when contemplating restoration repair as a treatment alternative to total replacement of restorations with localised imperfections [5, 8, 9]. The individual patient’s caries risk, the clinical condition and prognosis of the restored tooth unit and cost benefit impact assessments are the most critical and frequent ones. Broadly, the criteria for repair as opposed to total restoration replacement can be categorised as patient-centred and tooth-specific [5, 8, 9].

## Patient-Centred Criteria

Regular dental attendees, who are informed and dentally motivated, maintain a high standard of dental hygiene, and in whom the repaired restorations can be monitored on a regular basis, are ideal candidates for repair procedures. The other group of suitable patients for repair procedures are those with complicated medical histories or limited ability to cooperate with more extensive dental treatment. In such patients, the clinical intervention should be reduced in terms of duration and intricacy. Restoration refurbishment and repair procedures are particularly advantageous for patients with challenging medical histories and/or dental anxiety as the procedure can often be accomplished without the need to administer local anaesthesia. It is important that patients understand the nature of the repair procedure and how this procedure differs from restoration replacement. In order for patients’ consent for restoration refurbishment or repair procedures to be informed and valid, it is imperative to discuss the drawbacks of the replacement approach, regarding its effect on the prognosis of the repaired restoration and restored tooth unit. Similarly, the benefits of the repair approach, particularly in relation to preserving existing tooth structure, thereby not weakening the tooth, and its minimally interventional nature must be discussed.

The decision on the clinical management of a resin composite restoration with adjacent secondary caries for instance, should also be driven by the caries risk of the patient; whereby the decision to repair rather than replace is more likely to be correct in low and medium caries-risk patients. It must be noted that if the decision is made to replace the restoration in its entirety in a low or medium caries-risk patient, the cavity preparation will be enlarged unnecessarily, resulting in inappropriately weakening of the restored tooth. The resulting larger restoration is likely to be more prone to suffer uncertainties, including restoration failure, and the longevity of the restored tooth may be compromised and be at risk of more complex and expensive subsequent treatment, including endodontic treatment. It must be borne in mind that even when the restoration is replaced in its entirety, it may be subjected to the same, possibly unrecognised limitations as the original restoration. Thus, it is paramount to identify and, if possible, eliminate the factors that have contributed to the failing of the original restoration. In addition, repair procedures are likely to be less distressing for anxious patients when compared with replacement procedures, particularly when the patient understands that repair as opposed to replacement is contributing to them to ‘buy time’ in the quest to make restorations last as long as possible [33••].

## Tooth-Specific Criteria

In order to evaluate the tooth-specific criteria, it is essential to utilise an appropriate selection of clinical examination techniques providing that no single technique on its own would be sufficient to provide all the necessary information. For instance, magnification tools for visual examination and appropriate radiographic images are paramount in enhancing the sensitivity and specificity of the clinical judgement.

## Clinical Indications for Restoration Repair

### Secondary Caries

A secondary caries lesion adjacent to the restoration margin should be considered and treated as a new primary lesion [8, 10]. In common with all patients who present with a new caries lesion, preventive actions should be initiated, followed by operative intervention if the lesion is diagnosed to be active and progressing through dentine or when cavitation has occurred. Operative intervention should be minimally interventional coupled with partial replacement of that portion of the adjacent restoration which is affected and undermined by the caries. The portion of the existing restoration which exhibits no clinical or radiographic evidence of caries should be left in place, unless there is justified clinical indication to perform total restoration replacement with its various consequences [5].

### Marginal Defects and Marginal Staining

It must be emphasised that the presence of marginal defects does not necessarily indicate the presence of secondary caries. If limited, marginal defects can be simply managed using refinishing procedures. When minor marginal defects are observed in the occlusal surfaces of posterior composite restorations which are imperceptible to the patient, it is suggested that these are best monitored, with operative intervention being delayed until there is evidence of plaque accumulation, food stagnation or discoloration which may suggest active caries. Marginal defects and staining in anterior resin composite restorations are more challenging owing to their inherent tendency to pick up exogenous stain. Refinishing procedures coupled, where necessary, with refurbishment of the composite restoration are usually the most effective way to successfully manage such staining effectively. On the other hand, when the composite restoration is considerably penetrated with staining, total restoration replacement may be indicated to obtain the best possible aesthetic outcome.

### Superficial Colour Modification

In the event that an erroneous shade had been chosen for a previously placed composite restoration, shade correction may be performed by resurfacing using a different shade of composite material. Ideally, the same brand and type of composite material should be utilised as the composite substrate, provided this detail of the material is known to the dental practitioner.

### Bulk Fracture

Presentation of a bulk fracture of a composite restoration, particularly, soon after its placement, is usually indicative of an underlying aetiological reason, such as premature contacts or excessive occlusal loading, which must be diagnosed and eliminated to avoid recurring bulk fracture, or potentially fracture including remaining tooth tissue. However, when bulk fracture occurs in a restoration that has been in clinical function for several years, it is likely to be the result of stress fatigue within the composite material [5, 8]. Provided the bulk fracture includes less than half of the existing restoration, repair may be indicated; however, the integrity of the remaining part of the restoration should be carefully assessed [9].

### Fracture of Adjacent Tooth Structure

Fracture of tooth structure adjacent to a composite restoration could occur for several reasons, including occlusal parafunction, trauma or following detrimental polymerisation stresses during restoration placement [5]. Restoration repair could be indicated if the cause of the fracture is correctly identified and, as a result, the possibility of further fracture reduced, possibly through a preventative measure such as the provision of a protective mouthguard for a patient with bruxism.

### Wear of the Restoration

Wear of a composite restoration may be followed by passive eruption, overeruption of the opposing tooth or possibly tilting of the adjacent tooth or teeth. If the wear of the restoration is confined to the occlusal surface and subject to the availability of sufficient space for additional layer to perform a repair, the worn occlusal surface may be resolved by resurfacing [5, 8, 9]. If the proximal restoration surface is affected by wear and there is no space to restore the anatomic form of the restoration, then replacement should be considered.

## Contraindications for Repair

Typically, contraindications for repair may include (i) lack of patients’ willingness to accept a repair as an alternative to restoration replacement, (ii) irregular dental attenders, (iii) patients at high risk of developing caries, (iv) presence of extensive caries undermining more than half of the existing composite restoration and (v) failed previous restoration repair.

## Success of Repaired Restorations

Numerous longitudinal clinical studies have shown that restoration repairs in permanent teeth are able to significantly increase the lifetime of restorations and the restored tooth unit [10, 34–38] and come with reduced treatment time, lower costs and lower risks of complications than total replacements [28•]. The evidence from practice-based clinical studies indicated that repaired and replaced restorations showed similar survival outcomes regarding marginal defects and secondary caries in patients with low and medium caries risk, and most of the restorations were considered clinically acceptable after 3, 7, 10 and 12 years of clinical service [10, 34, 36, 38]. One prospective cohort practice-based study reported that teeth with repaired restorations were less likely to require endodontic treatment or extraction than those with replaced restorations over the 12-month observation period [39]. Clinical longevity of repairs is also highly dictated by the employed surface conditioning methods and meticulous application of the adhesive protocols that were extensively elaborated in a previous review [40••].

Whilst the placement of a glass ionomer based restorative material as either replacement of missing dental tissue or management of a defective restoration could be viewed as 'patching' that is of temporary nature, this should be followed, whenever possible, by a more definitive treatment—usually either in the form of restoration repair or replacement. Therefore, ‘patching’ refers rather to a short-term measure with limited longevity, whilst repair is a medium to long-term one. Certainly, not all repairs are the same as this is by and large, albeit not exclusively, dictated by the substrate type to be repaired along with the durability of adhesion achieved on the substrate and the location in the oral cavity in question [40••, 41••]. Currently, information is lacking in the dental literature as regards to the comparison of clinical longevity of a repaired restoration versus complete renewal such as a crown where the latter would surely require tooth preparation but would at the same time potentially survive shear forces during chewing function longer. Yet, possible loss of pulp vitality, caries and periodontal disease progression that are often associated with invasive full coverage reconstruction types [42] should be taken into consideration and communicated with the patients in clinical decision making between repair and replacement.

## Concluding Remarks

Reparative dentistry in terms of repairing defective restorations is a safe and effective treatment and should be viewed as a reliable alternative to increase the longevity of restorations and teeth, thereby avoiding an increase in cavity size and postponing the indication of more invasive treatments, such as crown restorations and, possibly, root canal treatments due to loss of tooth vitality. Considering numerous benefits of the repair treatment, this treatment modality should be indicated more often, especially in patients with low caries risk.
